# Detection of EGFR mutations at pM concentration in ten minutes using a microfluidic concentration and separation module

**DOI:** 10.1007/s10544-025-00767-w

**Published:** 2025-08-28

**Authors:** Jeffrey Teillet, Anne Pradines, Naima Hanoun, Jules Edwards, Pierre Joseph, Anne-Marie Gué, Aurélien Bancaud, Pierre Cordelier

**Affiliations:** 1 Centre de Recherches en Cancérologie de Toulouse, CRCT, Université de Toulouse, INSERM, CNRS, Toulouse, France; 2 LAAS-CNRS, Université de Toulouse, CNRS, Toulouse, France; 3 Laboratoire de Biologie Médicale Oncologique, Oncopole Claudius Regaud, IUCT- Oncopole, Toulouse, France

## Abstract

Epidermal growth factor receptor (EGFR) mutation detection is now commonly used in the management of cancer patients, particularly those diagnosed with non-small cell lung cancer. Molecular beacon-based sensing is direct and rapid, but its sensitivity is low. Conversely, high-sensitivity detection methodologies based on amplification are robust and sensitive but are limited by relatively require long turnaround times. In this study, we utilized a size-resolved, molecular beacon-based strategy for the rapid detection of EGFR genomic alterations, specifically exon 19 deletions and L858R point mutation. This technology combines a concentration and separation module, which allows us to successfully demonstrate the detection of deletions and point mutations of EGFR in five minutes with a mutant allele sensitivity of 10%. The use of a dual-color detection insures fast detection with a reduced risk of false positives. This work represents a first step toward the fast and specific detection of genetic mutations to improve the management of patients with hard-to-treat tumors.

**Supplementary Information:**

The online version contains supplementary material available at 10.1007/s10544-025-00767-w.

## Introduction

The epidermal growth factor receptor (EGFR) plays a critical role in regulating cell growth, survival, and differentiation (Sabbah et al. 2020). Mutations in the *EGFR* gene are frequently associated with a variety of cancers, particularly non-small cell lung cancer (NSCLC), and are key drivers of tumor progression (Da Cunha Santos et al. 2011). Detecting these mutations is vital for guiding targeted therapies, such as the tyrosine kinase inhibitors (TKIs) Erlotinib or Gefitinib (Cheng et al. 2023; Remon et al. 2018) and more recently Osimertinib (Ramalingam et al. 2020; Soria et al. 2018), which are highly effective only in patients with specific EGFR mutations. Identifying EGFR mutations may not only help select patients who are most likely to benefit from EGFR-targeted therapies (Karachaliou and Rosell 2014), but also improve the monitoring of the treatment response using e.g., liquid biopsies to detect changes in EGFR mutation status over time (Oh 2019), which may predict treatment outcomes and the development of drug resistance. Therefore, EGFR mutation detection is pivotal for the development of companion diagnostics, and requires precise, sensitive, and rapid technologies in both clinical and research settings.

As a matter of fact, advances in molecular biology and genomic technologies continue to enhance our ability to detect and interpret genetic mutations of EGFR in clinical routine. Traditional approaches, such as polymerase chain reaction (PCR)-based techniques, offer simplicity and speed but the standard implementation often lacks the sensitivity to detect low-frequency mutations in heterogeneous samples (Yang et al. 2021). Improvement for the detection of any mutation involving single base changes or small deletions were accomplished using the amplification-refractory mutation system (Little 1995), which detects mutant allele frequency of approximately 1% with real-time PCR. Digital droplet PCR (ddPCR) offers significantly higher sensitivity and is widely used in liquid biopsies with a mutant allele detection of 0.05% in clinical and research settings (Parkin 2019a). For genome-wide mutation analysis, sequencing technologies, particularly next-generation sequencing, have revolutionized mutation analysis, providing highly multiplexed mutation information of the *EGFR* gene with high sensitivity and throughput (Batra et al. 2022; Gao et al. 2016). However, the long time and high cost of these technologies remain practical issues for companion diagnostic tests, especially in high-risk patient populations or the general population with less access to medical tests.

The pursuit of high-performance detection methods that operate without molecular amplification, i.e., potentially enabling simpler implementation, remains an active area of research. Several studies have explored strand displacement amplification (Liu et al. 2020; Ma et al. 2024), an isothermal nucleic acid amplification technique that utilizes restriction enzymes and a strand-displacing DNA polymerase. When coupled to CRISPR, these methods reach high sensitivity of 0.1–1% mutant allele detection (Wang et al. 2021), but are generally considered less sensitive than ddPCR (Parkin 2019b). An alternative implementation of amplification-free detection commonly relies on mass, electrical, or optical index variation, achieving fM performance with 1–10% mutant allele sensitivity (Ansari et al. 2016; Ferrier et al. 2015; Jiang et al. 2021; Nesvet et al. 2021; Ondraskova et al. 2023). The highest sensitivities are generally obtained with nanosensors, though their response time is limited by slow target-probe interaction kinetics governed by diffusion (Squires et al. 2008). In contrast, bulk assays allow faster nucleic acid detection but at the cost of lower sensitivity. For instance, molecular beacon (MB) probes, which fluoresce upon hybridization to their target, can achieve a limit of detection (LoD) of 1 nM within 20–60 min (Baker et al. 2011). To balance speed and sensitivity, concentration modules have been introduced. By combining conventional MB probes with isotachophoresis, an LoD of 5 pM in just 3 min has been reported (Bahga et al. 2013; Bercovici et al. 2012; Persat and Santiago 2011).

Alternatively, we recently developed the µLAS technology (µ-Laboratory for DNA analysis and separation), which enables DNA size separation, concentration, and purification in a microchannel using bi-directional electrohydrodynamic actuation (Malbec et al. 2019; Milon et al. 2019, 2020; Ranchon et al. 2016). This technology consists in controlling the transport of DNA in a viscoelastic hydrodynamic flow with a counter electrophoretic force. It has been leveraged for nucleic acid detection by selectively concentrating target-MB complexes based on their greater size in comparison to unbound MB (Tijunelyte et al. 2021, 2022), achieving an LoD in the pM to fM range within 60 s. Here, we apply the µLAS platform to perform proof-of-concept EGFR point mutation and deletion detection with a mutant allele sensitivity of 10%. While the principle of the detection is unchanged, we use a two-color detection strategy with two MBs that target a control region and a mutated region in order to minimize false positives. We discuss the strengths and limitations of this assay in the context of companion diagnostics applications.

### Definition of the MB and their target sequences

**Fig. 1:**
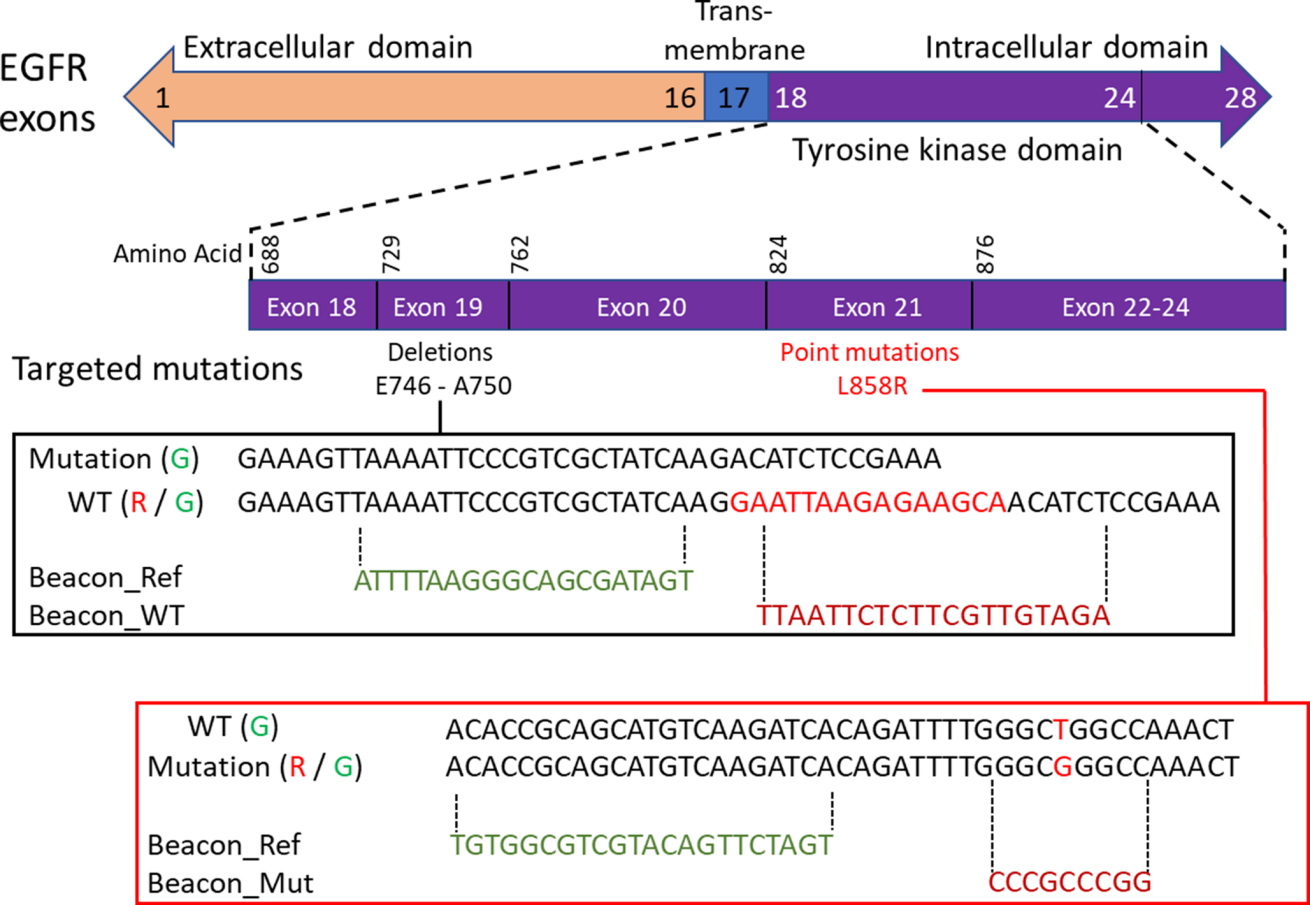
Genetic targets definition. The upper panel shows the structure of the EGFR gene. Frequent mutations in the intercellular domain occur in exon 19 and exon 21. We focus on the deletions in exon 19 and the single bp mutation in exon 21, as schematized by red sequences in the bottom panels. All the MBs are flanked by the GCGCGG

The TKI domain of EGFR contains several regions frequently mutated in cancer, with distinct mutation patterns across different exons. These mutations commonly include both point mutations and deletions (Sharma et al. 2007). In this study, we focus on exon 19 and exon 21, which are known mutation hotspots. Exon 19 is primarily affected by deletions of variable lengths, whereas exon 21 harbors a recurrent point mutation. This mutation results from a thymine-to-guanine substitution, leading to an amino acid change from leucine to arginine (highlighted in the red box in Fig. 1).

To detect this mutation, we use a MB (see sequences in Table 1) with a short 9-bp probe sequence (shown in dark red) that is specifically complementary to the mutated allele. Additionally, we target a conserved 22-nt reference sequence located upstream of the mutation site using another MB (depicted in green within the red box in Fig. 1). This reference MB hybridizes with both mutated and wild type (WT) fragments, providing a control signal. By measuring fluorescence signals from the green (reference) and red (mutation-specific) MBs in separate detection channels, we can simultaneously quantify the mutated and WT sequences. This dual-probe approach ensures the accurate detection of the mutation by preventing false negatives that could arise from a failure to detect the mutation-specific signal alone. If only the reference signal is present without the mutation signal, it confirms the presence of WT sequences, distinguishing true negatives from technical failures.

For exon 19 deletions, ten distinct variants have been reported between positions K745 and K754, spanning approximately 30 base pairs (Sharma et al. 2007). To detect these, we employ a drop-off method, which is commonly used in clinical diagnostics (black box in Fig. 1; (Decraene et al. 2018). This method specifically targets the WT sequence and is adapted to detect multiple deletions clustered within a narrow genomic region. Rather than employing a separate probe for each possible deletion, any mutation within the target region leads to a loss of signal. In our system, the WT allele spans 55 nucleotides, while the deletion—highlighted in red—removes 15 nucleotides. The drop-off probe loses fluorescence in the presence of a deletion, while a reference probe (shown in green) generates a signal that reflects the combined amount of WT and deleted molecules. This persistent reference signal not only confirms the presence of a deletion but also reduces the likelihood of false negatives.

### Design and characterization of the MB

The MBs were designed by flanking the complementary sequences, as shown in Fig. 1, with a GC-rich stem (CCGCGC). The stem’s melting temperature was 24 °C, slightly above room temperature in order to balance the stability of the closed (quenched) configuration with the open configuration upon hybridization with the target sequence.

**Fig. 2:**
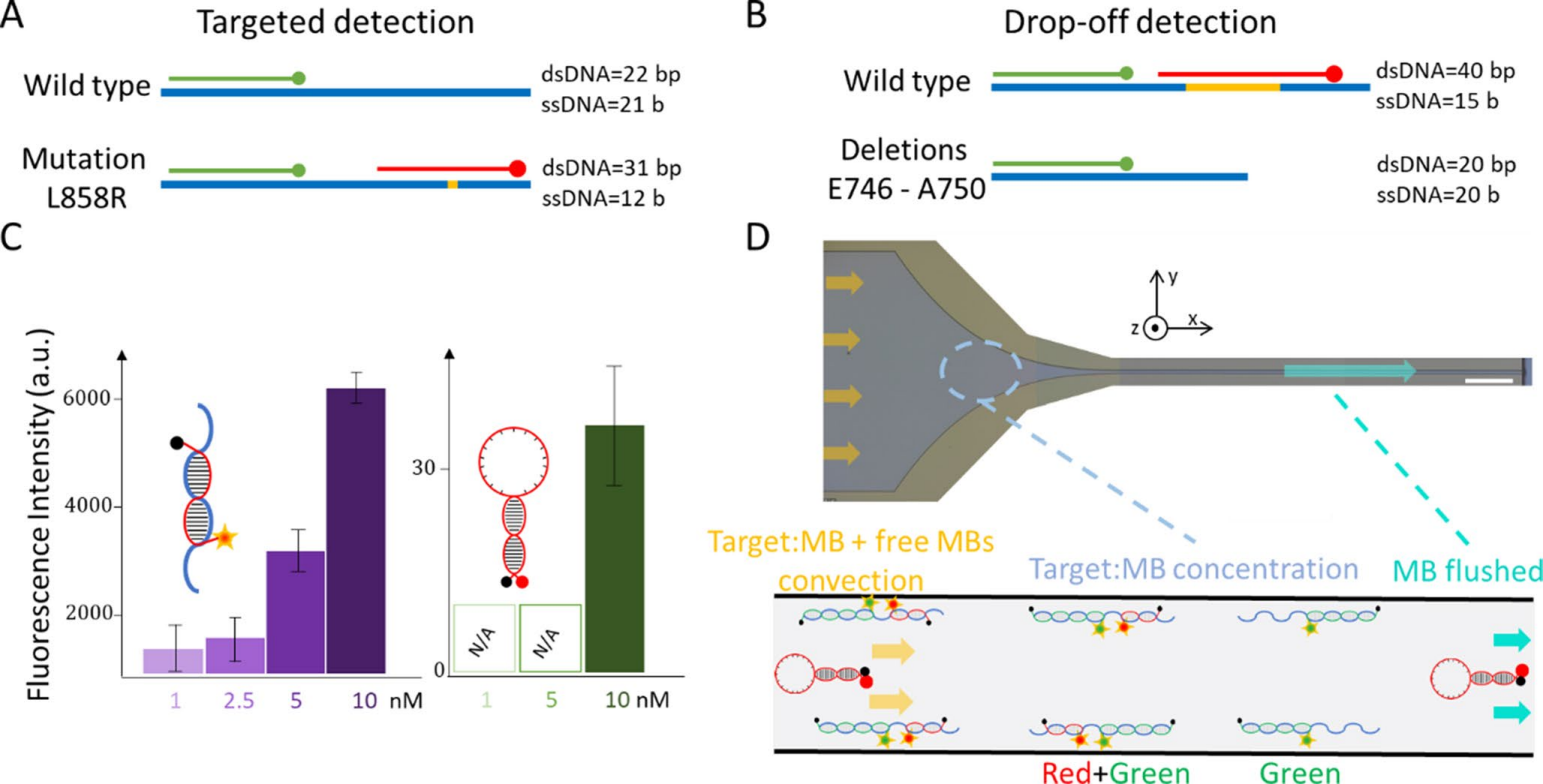
Molecular beacon definition and principle of the detection.(**A**) The schemes depict the WT and mutated sequence with MBs and the size in bp of the resulting complex in the targeted mode of detection. (**B**) Same as (A) in the drop-off detection strategy. (**C**) The plot on the left shows fluorescence intensity as a function of target concentration at a MB concentration of 5 nM. As illustrated in the plot on the right, the fluorescence of unbound MBs is negligible compared to that of MB-target complexes. (**D**) The micrograph represents the microfluidic concentration module with the shape of a funnel (scale bar = 100 μm). The bottom panel sketches the result of the operation of µLAS with the concentration of the target with one or two MBs at different positions and the flushing of unbound MBs

In the direct mutation detection mode (Fig. 2A), the reference MB formed a 22-base-pair double helix with both the wild-type (WT) and mutated sequences (green segment). The mutation-specific MB, however, contained a 9-nucleotide segment centered around the mutation hotspot (red segment), resulting in a total double-stranded DNA length of 31 base pairs (bp). This design was chosen to ensure high selectivity of MB binding to the mutated sequence while maintaining specificity. A similar strategy was used for drop-off detection, which consisted in targeting a 20-bp reference fragment upstream of the mutated region and another 20-bp sequence only present in the WT EGFR (Fig. 2B).

Bulk fluorimetry measurements confirmed that the MB was effectively quenched in the absence of its complementary sequence (Fig. 2C). Upon addition of the target sequence, a detectable fluorescence signal of 710 ± 460 arbitrary units (a.u.) was observed at 1 nM, compared to 36 ± 11 a.u. for the MB alone. These results confirm that MBs enable sub-nanomolar detection, consistent with previous findings (Baker et al. 2011).

We aimed to combine the detection of MB with the µLAS separation and concentration module. The principle of this approach consists in using electrohydrodynamic actuation in a viscoelastic fluid in order to induce transverse migration oriented toward the upper and lower channel walls. The transverse migration force increases with hydrodynamic flow velocity, electrophoretic velocity, and DNA molecular weight (MW) (Chami et al. 2018, 2020). The latter dependence allows us to perform DNA separation in a linear channel, i.e. with constant hydrodynamic and electrophoretic settings (Ranchon et al. 2016). In a microchannel with the shape of a funnel (micrograph in Fig. 2D), the flow velocity and electric field build up near the constriction due to flux conservation, and so does the amplitude of the transverse force. By adjusting the pressure and tension actuation settings, hydrodynamic and electrophoretic forces can be balanced at a defined position of the funnel where the DNA migration velocity is null. Upstream of this stagnation position, hydrodynamic forces prevail whereas electrophoretic forces direct a motion in the opposite direction past the stagnation point. Consequently, DNA molecules continuously flow to and accumulate at this position. Furthermore, the concentration process is size-dependent because transverse forces depend on the MW of the DNA. This property enables us to detect the molecules with one or two MBs at separate position, and it allows us to flush out single stranded molecules, which are not arrested in the funnel (Tijunelyte et al. 2021). Using this technology, we thus simultaneously concentrate and separate target: MB complexes, and selectively wash out unreacted MB, which are the smaller objects with a dsDNA stem of 6 bp (cyan arrow in Fig. 2D). This distinctive feature, which allows us to detect a target with a simultaneous quality control check test, is investigated with the EGFR probes for drop-off and targeted detection.

### EGFR mutation detection

We performed experiments with MBs for targeted and drop-off detection of EGFR mutation, as defined in Fig. 1. We prepare a mix of the WT and mutant sequences at a concentration ratio of 1:10, using respective concentrations of 1 and 0.1 nM (see methods for details). The MBs for the reference and target mutation sequences were added to the mix at the same concentration of 1 nM. The sample was injected in the chip of 2 μm in thickness (see methods for details) and electrohydrodynamic actuation was triggered with 4 bar in pressure and 210–180 V in tension for the targeted and drop-off detection, respectively, which corresponded to maximum flow velocity and electric fields at the funnel of 20 mm/s and typically 15 kV/cm (Tijunelyte et al. 2022). Note that the smaller MW of the target: MB complex in targeted vs. drop-off detection (see Fig. 2A) requires larger electrohydrodynamic transverse forces, explaining the use of a higher electric field for the targeted detection strategy. As a starting point, we performed the same characterization as in our previous report (Tijunelyte et al. 2022), and characterized the concentration process using a linear target and one MB (reference sequence in Fig. 1 without the stem motif). The accumulation kinetic of the target: MB complex was independent of the probe concentration and equal to 3.3-fold per minute on average during the first 400 s (Supplementary Fig. S1A-B). Setting the time window of acquisition to 350 s, we determined the limit of detection of the target: MB to 770 +/- 230 fM (Supplementary Fig. S1C-D).

**Fig. 3:**
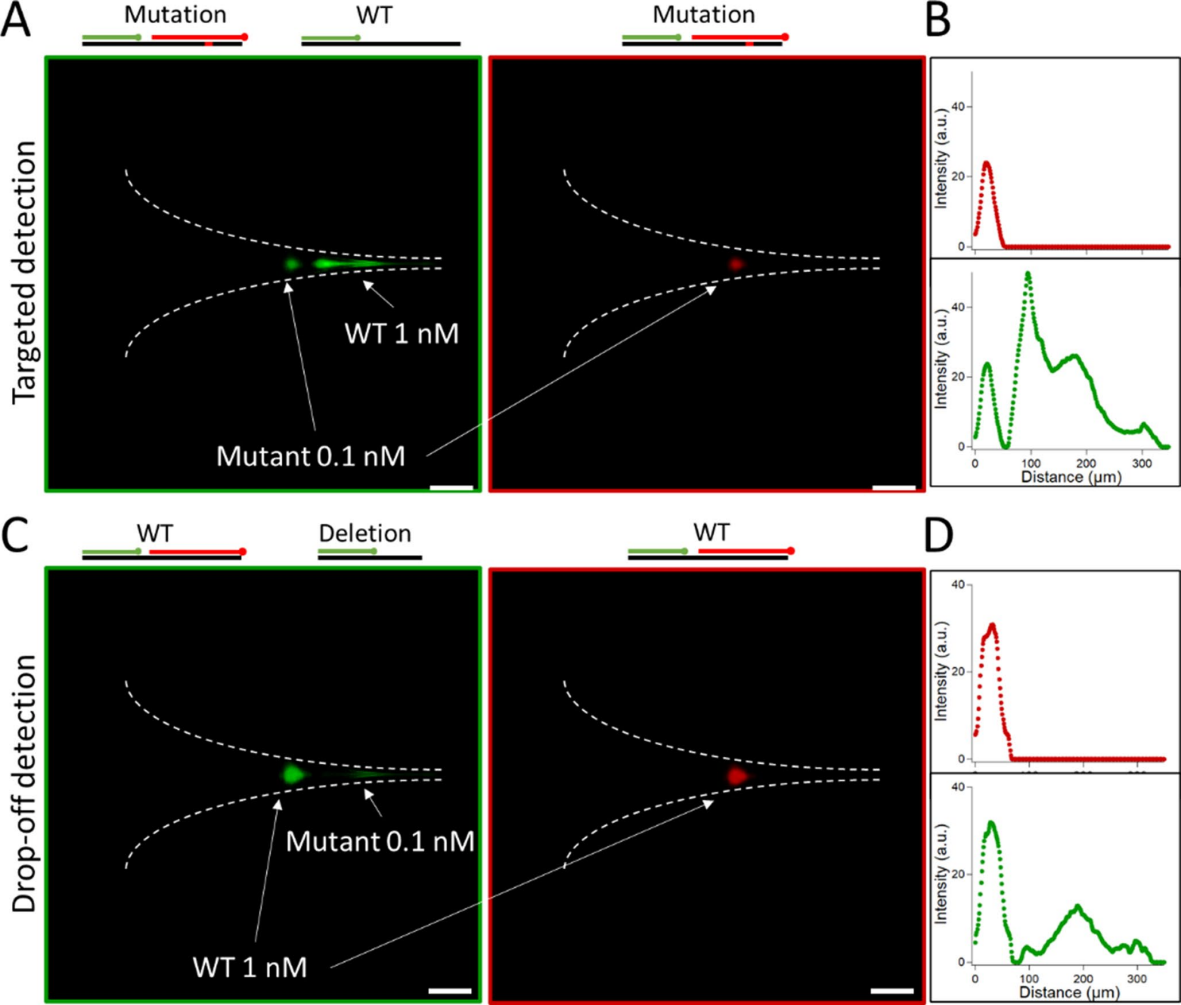
EGFR mutation detection.(**A**) The two fluorescence micrographs represent the signal collected in the funnel (dashed white lines) after three minutes of concentration using 488 nm and 560 nm excitation (left and right images, respectively). (**B**) The two plots represent the fluorescence intensity profile along the horizontal symmetry axis of the microfluidic chip in the green and the red channel, as outlined on the two images of panel (A). (**C**) Same as panel (A) in the drop off detection technology. (**D**) Same as (B) for the two images of panel (C). See the accompanying videos in Supplementary Material

We then proceeded to the detection of EGFR mutations, specifically focusing on point mutations at first. Using a video camera, we recorded signals in the red and green channels via the targeted detection method (Fig. 3A). Our observations revealed two distinct patterns in the green channel but only one in the red channel (Fig. 3A; see the single peak signal in the red channel inset). This outcome aligns with the expected behavior: the red channel exclusively detects the mutated sequence, whereas the green channel captures both the WT and mutated fragments (as depicted in the upper panel of Fig. 3A). Additionally, the spatial superposition of green and red spots was anticipated, given that high-molecular-weight (MW) molecules accumulated in both channels. Notably, in the green channel, the low-MW spot, located closer to the apex of the funnel, exhibited a higher intensity than the high-MW spot (Fig. 3B). By computing the spatial integral of the two signals, we determined that the low-MW spot was 7.3 times more concentrated. This result is consistent with the fact that the reference signal, which consists of a complex with one MB, is 10-fold more concentrated. These findings confirm that µLAS technology effectively concentrates and spatially separates the reference and mutated sequences, enabling clear and unambiguous identification.

Next, we repeated the experiment using the drop-off strategy. Similar to the previous approach, we observed two peaks in the green channel and only one in the red channel (Fig. 3B), with the high-MW band appearing spatially coincident in both channels. However, in this case, the low-MW spot was less intense than the high-MW spot, and the concentration ratio, determined via spatial integration, yielded a high: low-MW ratio of 0.3-fold. These results align with the expected behavior: the reference signal, which forms a complex with two MBs, has a higher MW (as illustrated in the upper panel of Fig. 3B). The mutant sequence appears as a low-MW spot, i.e., close to the apex of the funnel, at a lower concentration (Fig. 3D). This detection setup confirms that separation and detection effectively identify mutations with no risk of false negatives.

## Discussion

During this study, we demonstrated that gene mutations relevant to the management of hard-to-treat tumors can be detected in 300 s with high sensitivity. We employed a spatially resolved MB-based detection method, which proved instrumental in distinguishing mutant from WT signals, enabling dual detection in a single reaction. This not only saves time and preserves valuable clinical samples but also enhances the reliability of the method by considering both specificity and sensitivity. However, our study has several limitations. First, we report a mutant allele frequency of 10%, which is significantly higher than that reported for ddPCR (Dong et al., 2018). This limit could be improved by refining the probes (size, sequence, and dyes with improved photostability and quantum yield) and/or optimizing the detection system, for example, by using more sensitive cameras, high numerical aperture objectives. Nonetheless, our approach offers a rapid screening strategy to identify patients with the highest tumor burden, thereby facilitating earlier clinical intervention—a factor often critical to therapeutic success. An exciting extension of this work would be to explore other clinically-relevant EGFR point mutations. Future developments, such as incorporating multiple probe sizes to take advantage of the spatial separation and using more spectrally-separated dyes, could enable the simultaneous detection of e.g., S768I, T790M, L858R, L861Q, G719X, and C797S in a single reaction, further reducing time and sample requirements. Since we used artificial probes in this study, the next step will be to validate this approach by detecting EGFR gene deletions or mutations in cell extracts and in plasma from patients with EGFR-mutated tumors, such as NSCLC, a feature that is unique to the µLAS system and cannot be replicated by conventional amplification strategies.

## Conclusion

We present a novel application of µLAS technology for EGFR mutation detection, specifically targeting exon 19 and exon 21, which exhibit distinct mutational patterns—multiple deletions and single point mutations, respectively. For the first time using this platform, we implement a two-color detection strategy that simultaneously senses both a reference sequence and a mutation, reducing the risk of false negatives. Leveraging the µLAS system’s integrated size separation and concentration capabilities, our approach enables rapid detection with minimal reagent consumption. These advantages position the technology as a promising tool to support faster clinical decision-making while remaining accessible, cost-effective, and adaptable for both population-level screening and ongoing cancer risk monitoring.

## Materials and methods

### Reagents

Chemicals were purchased from Signa-Aldrich, unless mentioned. The neutral polymer polyvinylpyrrolidone (PVP; MW = 1.3 MDa) was dissolved in water at 6% (w: v) concentration. This stock solution was mixed with PBS (Dulbecco’s Phosphate buffer saline) at a final PVP concentration of 3% adding 22 mM of NaCl in order to increase the fluorescence signal in comparison to electrophoresis buffers (see ref. (Tijunelyte et al. 2021). The viscosity of the solution were 20 mPa.s, respectively (Chami et al. 2018).

### Genomic material

Eight oligonucleotides were purchased from Eurogentec (Belgium), dissolved in Tris-HCl 10 mM and EDTA 1 mM (pH = 7.5) at a stock concentration of 100 µM, and stored at 20 °C. The green and red fluorophores were 6-carboxyfluorescein and hexachlorofluorescein, respectively. We used the same Minor Groove Binder/Elipse Dark Quencher for both fluorophores. Nucleic acid samples were prepared as follows: (i) the stock of probe and target were diluted in 1X-DPBS at 1 µM; (ii) 1 µL of target and 1 µL of the probe were then mixed, heated at 92 °C for 5 min, and cooled down to RT; (iii) PVP based buffer was added to obtain the final probe: target complex concentration. The solution was then extensively vortexed for three minutes and left at room temperature to homogenize for one hour.

### Microfluidic chip fabrication and operation

The microfluidic chips with a uniform height of 2 μm were obtained by conventional photolithography with the protocol detailed in ref. (Chami et al. 2018) (see details of the geometry in Supplementary Fig. S2). We used 4-inch silicon wafers that were processed with a first step of reactive ion etching down to a depth of 2 μm (Supplementary Fig. S3A-B). We performed a second step of photolithography and etching to produce thicker access channels of 10 μm. We drilled access holes through the silicon wafer by sand blasting, and finally performed dry oxidation to grow an insulating layer of 200 nm in thickness. The processed wafer was sealed to 160 μm glass 4-inch wafer by anodic bonding. The wafers were eventually sliced and diced with a diamond saw into individual chips.

Fabricated chips were then placed in a custom chip support with inlet and outlet reservoirs sealed by O-rings ((Tijunelyte et al. 2021), Supplementary Fig. S3C). The reservoirs contained 0.5 mm diameter platinum wires (GoodFellow) as electrodes. Actuation was operated with a 7 bar pressure controller (Fluigent) and a 200 V DC power supply (Labsmith). Before each experiment, microfluidic chips were filled with ethanol to eliminate air bubbles, then flushed with milli-Q water, and subsequently filled with the PVP working buffer. Data were acquired using an Olympus epifluorescence microscope equipped with a sCMOS digital camera (Hamamatsu, Supplementary Fig. S3D). 20X air objective (NA = 0.8) was used. For time-lapse video acquisitions, the inter-frame interval was set to 1 s and a binning of 4 × 4 (512*152 pixels of 1.3 μm in size).

## Electronic supplementary material

Below is the link to the electronic supplementary material.

Supplementary Material 1

## Data Availability

No datasets were generated or analysed during the current study.

## References

[CR1] M.I.H. Ansari, S. Hassan, A. Qurashi, F.A. Khanday, Microfluidic-integrated DNA nanobiosensors. Biosens. Bioelectron. **85**, 247–260 (2016). 10.1016/j.bios.2016.05.009 27179566 10.1016/j.bios.2016.05.009

[CR2] S.S. Bahga, C.M. Han, J.G. Santiago, Integration of rapid DNA hybridization and capillary zone electrophoresis using bidirectional isotachophoresis. Analyst. **138**, 87–90 (2013). 10.1039/C2AN36249J 23103998 10.1039/c2an36249j

[CR3] M.B. Baker, G. Bao, C.D. Searles, In vitro quantification of specific MicroRNA using molecular beacons. Nucleic Acids Res. Gkr. 1016 (2011). 10.1093/nar/gkr1016 10.1093/nar/gkr1016 PMC3258119 22110035

[CR4] U. Batra, S. Nathany, M. Sharma, P. Jain, A. Mehta, Next generation sequencing for detection of *EGFR* alterations in NSCLC: is more better? J. Clin. Pathol. **75**, 164–167 (2022). 10.1136/jclinpath-2020-207212 33372105 10.1136/jclinpath-2020-207212

[CR5] M. Bercovici, C.M. Han, J.C. Liao, J.G. Santiago, Rapid hybridization of nucleic acids using isotachophoresis. Proc Natl Acad Sci **109**, 11127–11132 (2012). 10.1073/pnas.1205004109 22733732 10.1073/pnas.1205004109 PMC3396536

[CR6] B. Chami, M. Socol, M. Manghi, A. Bancaud, Modeling of DNA transport in viscoelastic electro-hydrodynamic flows for enhanced size separation. Soft Matter. **14**, 5069–5079 (2018). 10.1039/c8sm00611c 29873390 10.1039/c8sm00611c

[CR7] B. Chami, N. Milon, F. Rojas, J.-L. Charlot, S. Marrot, J.-C. Bancaud, A, Single-step electrohydrodynamic separation of 1–150 Kbp in less than 5 min using homogeneous glass/adhesive/glass microchips. Talanta. **217**, 121013 (2020). 10.1016/j.talanta.2020.121013 32498826 10.1016/j.talanta.2020.121013

[CR8] Z. Cheng, H. Cui, Y. Wang, J. Yang, C. Lin, X. Shi, Y. Zou, J. Chen, X. Jia, L. Su, The advance of the third–generation EGFR–TKI in the treatment of non–small cell lung cancer (Review). Oncol. Rep. **51**, 16 (2023). 10.3892/or.2023.8675 38063215 10.3892/or.2023.8675 PMC10739988

[CR9] Da G. Cunha Santos, F.A. Shepherd, M.S. Tsao, EGFR mutations and lung Cancer. Annu. Rev. Pathol. Mech. Dis. **6**, 49–69 (2011). 10.1146/annurev-pathol-011110-130206 10.1146/annurev-pathol-011110-130206 20887192

[CR10] C. Decraene, A.B. Silveira, F.-C. Bidard, A. Vallée, M. Michel, S. Melaabi, A. Vincent-Salomon, A. Saliou, A. Houy, M. Milder, O. Lantz, M. Ychou, M.G. Denis, J.-Y. Pierga, M.-H. Stern, C. Proudhon, Multiple hotspot mutations scanning by single droplet digital PCR. Clin. Chem. **64**, 317–328 (2018). 10.1373/clinchem.2017.272518 29122835 10.1373/clinchem.2017.272518

[CR11] D.C. Ferrier, M.P. Shaver, P.J.W. Hands, Micro- and nano-structure based oligonucleotide sensors. Biosens. Bioelectron. **68**, 798–810 (2015). 10.1016/j.bios.2015.01.031 25655465 10.1016/j.bios.2015.01.031

[CR12] J. Gao, H. Wu, X. Shi, Z. Huo, J. Zhang, Z. Liang, Comparison of Next-Generation sequencing, quantitative PCR, and Sanger sequencing for mutation profiling of EGFR, KRAS, PIK3CA and BRAF in clinical lung tumors. Clin. Lab. 62 (2016). 10.7754/Clin.Lab.2015.150837 10.7754/clin.lab.2015.150837 27215089

[CR13] H. Jiang, H. Xi, M. Juhas, Y. Zhang, Biosensors for point mutation detection. Front. Bioeng. Biotechnol. **9**, 797831 (2021). 10.3389/fbioe.2021.797831 34976987 10.3389/fbioe.2021.797831 PMC8714947

[CR14] N. Karachaliou, R. Rosell, Targeted treatment of mutated EGFR-expressing non-small-cell lung cancer: focus on erlotinib with companion diagnostics. Lung Cancer Targets Ther. 73 (2014). 10.2147/LCTT.S50671 10.2147/LCTT.S50671 PMC5217512 28210145

[CR15] S. Little, Amplification-Refractory mutation system (ARMS) analysis of point mutations. Curr. Protoc. Hum. Genet. **7** (1995). 10.1002/0471142905.hg0908s07 10.1002/0471142905.hg0908s07 18428319

[CR16] N. Liu, X. Zhang, X. Tang, Y. Liu, D. Huang, X. Xiao, A double-stranded DNA catalyzed strand displacement system for detection of small genetic variations. Chem. Commun. **56**, 14397–14400 (2020). 10.1039/D0CC06216B 10.1039/d0cc06216b 33140767

[CR17] Z. Ma, J. Xu, W. Hou, Z. Lei, T. Li, W. Shen, H. Yu, C. Liu, J. Zhang, S. Tang, Detection of single nucleotide polymorphisms of Circulating tumor DNA by strand displacement amplification coupled with liquid chromatography. Anal. Chem. **96**, 5195–5204 (2024). 10.1021/acs.analchem.3c05500 38520334 10.1021/acs.analchem.3c05500

[CR18] R. Malbec, B. Chami, L. Aeschbach, G.A. Ruiz Buendía, M. Socol, P. Joseph, T. Leïchlé, E. Trofimenko, A. Bancaud, V. Dion, µLAS: sizing of expanded trinucleotide repeats with femtomolar sensitivity in less than 5 minutes. Sci. Rep. **9**, 23 (2019). 10.1038/s41598-018-36632-5 30631115 10.1038/s41598-018-36632-5 PMC6328573

[CR19] N. Milon, C. Chantry-Darmon, C. Satge, M.-A. Fustier, S. Cauet, S. Moreau, C. Callot, A. Bellec, T. Gabrieli, L. Saïas, µLAS technology for DNA isolation coupled to Cas9-assisted targeting for sequencing and assembly of a 30 kb region in plant genome. Nucleic Acids Res. **47**, 8050–8060 (2019) 31505675 10.1093/nar/gkz632 PMC6736094

[CR20] N. Milon, F. Rojas, J.-L. Castinel, A. Bigot, L. Bouwmans, G. Baudelle, K. Boutonnet, A. Gibert, A. Bouchez, O. Donnadieu, C. Ginot, F. Bancaud, A, A tunable filter for high molecular weight DNA selection and linked-read sequencing. Lab. Chip. **20**, 175–184 (2020). 10.1039/c9lc00965e 31796946 10.1039/c9lc00965e

[CR21] J.C. Nesvet, K.A. Antilla, D.S. Pancirer, A.X. Lozano, J.S. Preiss, W. Ma, A. Fu, S.-M. Park, S.S. Gambhir, A.C. Fan, J.W. Neal, S.K. Padda, M. Das, T. Li, H.A. Wakelee, S.X. Wang, Giant magnetoresistive nanosensor analysis of Circulating tumor DNA epidermal growth factor receptor mutations for diagnosis and therapy response monitoring. Clin. Chem. **67**, 534–542 (2021). 10.1093/clinchem/hvaa307 33393992 10.1093/clinchem/hvaa307 PMC12118574

[CR22] I.-J. Oh, Can liquid biopsy-guided EGFR-targeted therapy be a surrogate for the tissue-based standard approach? Transl. Lung Cancer Res. **8**, S351–S354 (2019). 10.21037/tlcr.2019.06.03 10.21037/tlcr.2019.06.03 PMC6987342 32038913

[CR23] K. Ondraskova, R. Sebuyoya, L. Moranova, J. Holcakova, P. Vonka, R. Hrstka, M. Bartosik, Electrochemical biosensors for analysis of DNA point mutations in cancer research. Anal. Bioanal Chem. **415**, 1065–1085 (2023). 10.1007/s00216-022-04388-7 36289102 10.1007/s00216-022-04388-7

[CR24] B. Parkin, Rare variant quantitation using droplet digital PCR, in *Chronic Lymphocytic Leukemia, Methods in Molecular Biology*, ed. by S.N. Malek (Springer New York, New York, NY, 2019a), pp. 239–251. 10.1007/978-1-4939-8876-1_18 10.1007/978-1-4939-8876-1_18 30350210

[CR25] A. Persat, J.G. Santiago, MicroRNA profiling by simultaneous selective isotachophoresis and hybridization with molecular beacons. Anal. Chem. **83**, 2310–2316 (2011). 10.1021/ac103225c 21329391 10.1021/ac103225c

[CR26] S.S. Ramalingam, J. Vansteenkiste, D. Planchard, B.C. Cho, J.E. Gray, Y. Ohe, C. Zhou, T. Reungwetwattana, Y. Cheng, B. Chewaskulyong, R. Shah, M. Cobo, K.H. Lee, P. Cheema, M. Tiseo, T. John, M.-C. Lin, F. Imamura, T. Kurata, A. Todd, R. Hodge, M. Saggese, Y. Rukazenkov, J.-C. Soria, Overall survival with osimertinib in untreated, *EGFR* -Mutated advanced NSCLC. N Engl. J. Med. **382**, 41–50 (2020). 10.1056/NEJMoa1913662 31751012 10.1056/NEJMoa1913662

[CR27] H. Ranchon, R. Malbec, V. Picot, A. Boutonnet, P. Terrapanich, P. Joseph, T. Leïchlé, A. Bancaud, DNA separation and enrichment using electro-hydrodynamic bidirectional flows in viscoelastic liquids. Lab. Chip. **16**, 1243–1253 (2016). 10.1039/c5lc01465d 26936389 10.1039/c5lc01465d

[CR28] J. Remon, C.E. Steuer, S.S. Ramalingam, E. Felip, Osimertinib and other third-generation EGFR TKI in EGFR-mutant NSCLC patients. Ann. Oncol. **29**, i20–i27 (2018). 10.1093/annonc/mdx704 29462255 10.1093/annonc/mdx704

[CR29] D.A. Sabbah, R. Hajjo, K. Sweidan, Review on epidermal growth factor receptor (EGFR) structure, signaling pathways, interactions, and recent updates of EGFR inhibitors. Curr. Top. Med. Chem. **20**, 815–834 (2020). 10.2174/1568026620666200303123102 32124699 10.2174/1568026620666200303123102

[CR30] S.V. Sharma, D.W. Bell, J. Settleman, D.A. Haber, Epidermal growth factor receptor mutations in lung cancer. Nat. Rev. Cancer. **7**, 169–181 (2007). 10.1038/nrc2088 17318210 10.1038/nrc2088

[CR31] J.-C. Soria, Y. Ohe, J. Vansteenkiste, T. Reungwetwattana, B. Chewaskulyong, K.H. Lee, A. Dechaphunkul, F. Imamura, N. Nogami, T. Kurata, I. Okamoto, C. Zhou, B.C. Cho, Y. Cheng, E.K. Cho, P.J. Voon, D. Planchard, W.-C. Su, J.E. Gray, S.-M. Lee, R. Hodge, M. Marotti, Y. Rukazenkov, S.S. Ramalingam, Osimertinib in untreated *EGFR* -Mutated advanced Non–Small-Cell lung Cancer. N Engl. J. Med. **378**, 113–125 (2018). 10.1056/NEJMoa1713137 29151359 10.1056/NEJMoa1713137

[CR32] T.M. Squires, R.J. Messinger, S.R. Manalis, Making it stick: convection, reaction and diffusion in surface-based biosensors. Nat. Biotechnol. **26**, 417–426 (2008). 10.1038/nbt1388 18392027 10.1038/nbt1388

[CR33] I. Tijunelyte, R. Malbec, B. Chami, J. Cacheux, C. Dez, T. Leichlé, P. Cordelier, A. Bancaud, micro-RNA 21 detection with a limit of 2 pM in 1 min using a size-accordable concentration module operated by electrohydrodynamic actuation. Biosens. Bioelectron. **178**, 112992 (2021). 10.1016/j.bios.2021.112992 33548653 10.1016/j.bios.2021.112992

[CR34] I. Tijunelyte, J. Teillet, P. Bruand, R. Courson, A. Lecestre, P. Joseph, A. Bancaud, Hybridization-based DNA biosensing with a limit of detection of 4 fM in 30 s using an electrohydrodynamic concentration module fabricated by grayscale lithography. Biomicrofluidics. **16**, 044111 (2022). 10.1063/5.0073542 35992636 10.1063/5.0073542 PMC9385222

[CR35] M. Wang, D. Han, J. Zhang, R. Zhang, J. Li, High-fidelity detection of DNA combining the CRISPR/Cas9 system and hairpin probe. Biosens. Bioelectron. **184**, 113212 (2021). 10.1016/j.bios.2021.113212 33862567 10.1016/j.bios.2021.113212

[CR36] Z. Yang, W. Chen, J. Wang, M. Shi, R. Zhang, S. Dai, T. Wu, M. Zhao, Programmable One-Pot enzymatic reaction for direct fluorescence detection of Ultralow-Abundance mutations in the DNA duplex. Anal. Chem. **93**, 7086–7093 (2021). 10.1021/acs.analchem.1c00564 33901400 10.1021/acs.analchem.1c00564

