# Supplementary material for: Detection of EGFR mutations at pM concentration in ten minutes using a microfluidic concentration and separation module
Source: Biomed Microdevices. 2025 Aug 28;27(3):40. doi: 10.1007/s10544-025-00767-w (PMC12394390; doi:10.1007/s10544-025-00767-w)
Supplement: Supplementary file 1 — Supplementary Material 1 [file 10544_2025_767_MOESM1_ESM.docx]

Detection of EGFR Mutations at pM Concentration in Ten Minutes Using a Microfluidic Concentration and Separation Module

Jeffrey Teillet^1^, Anne Pradines^1^, Naima Hanoun^1^, Aurélien Bancaud^2^, Anne-Marie Gué^2^, Pierre Cordelier^1^

^1^ Centre de Recherches en Cancérologie de Toulouse, CRCT, Université de Toulouse, INSERM, CNRS, Toulouse, France.

^2^ LAAS-CNRS, Université de Toulouse, CNRS, Toulouse, France.

**Supplementary Video:**

**Media_drop_off.avi**

The green signal is recorded until 10:41, and the dichroic filter is then switched to record the red signal.

**Media_mutation.avi**

The green signal is recorded until 10:37, and the dichroic filter is then switched to record the red signal.

Calculation of the hydraulic resistance of the chip and flow rate:

The hydraulic resistance $R_{h}$ of a channel with a rectangular cross-section is given by

$R_{h}=\frac{12\mu L}{wh^{3}(1-0.63\frac{h}{w})}$ (S1)

where $\mu$ is the viscosity, $L$ is the length of the channel, $w$ its width and $h$ its height. In the funnel region, the width of the channel is follows a power-law response characterized by an exponent of 3. Therefore, the hydraulic resistance of this section of the channel is:

$R_{h}=\int_{0}^{500} \frac{12\mu}{(5+\left( \frac{x}{80} \right)^{3})h^{3}(1-0.63\frac{h}{5+\left( \frac{x}{80} \right)^{3}})}dx$ (S2)

The total hydraulic resistance is therefore the sum of equation (S1) for the four linear sections and equation (S2) for the funnel. We can then deduce the flow rate provided the definition of the hydraulic resistance $Q=\frac{\Delta P}{R_{h}}$:

$v_{max}\left( x \right)=\frac{3\Delta P}{2h(5+\left( \frac{x}{80} \right)^{3})R_{h}}$ (S3)

Calculation of the hydraulic resistance of the chip:

In a rectangular microchannel, the electric field is constant, and the electrical resistance is readily computed as

$R=\frac{\rho L}{wh}$ (S4)

Where $\rho$ is the conductivity equal of the buffer solution. The resistance of the funnel is equal to

$R=\int_{0}^{500} \frac{\rho dx}{h(5+\left( \frac{x}{80} \right)^{3}}$ (S5)

The continuity equation of the current allows us to determine the potential for each interface of the microchip and therefore the electric field provided that

$E=\frac{\Delta U}{L}$ (S6)


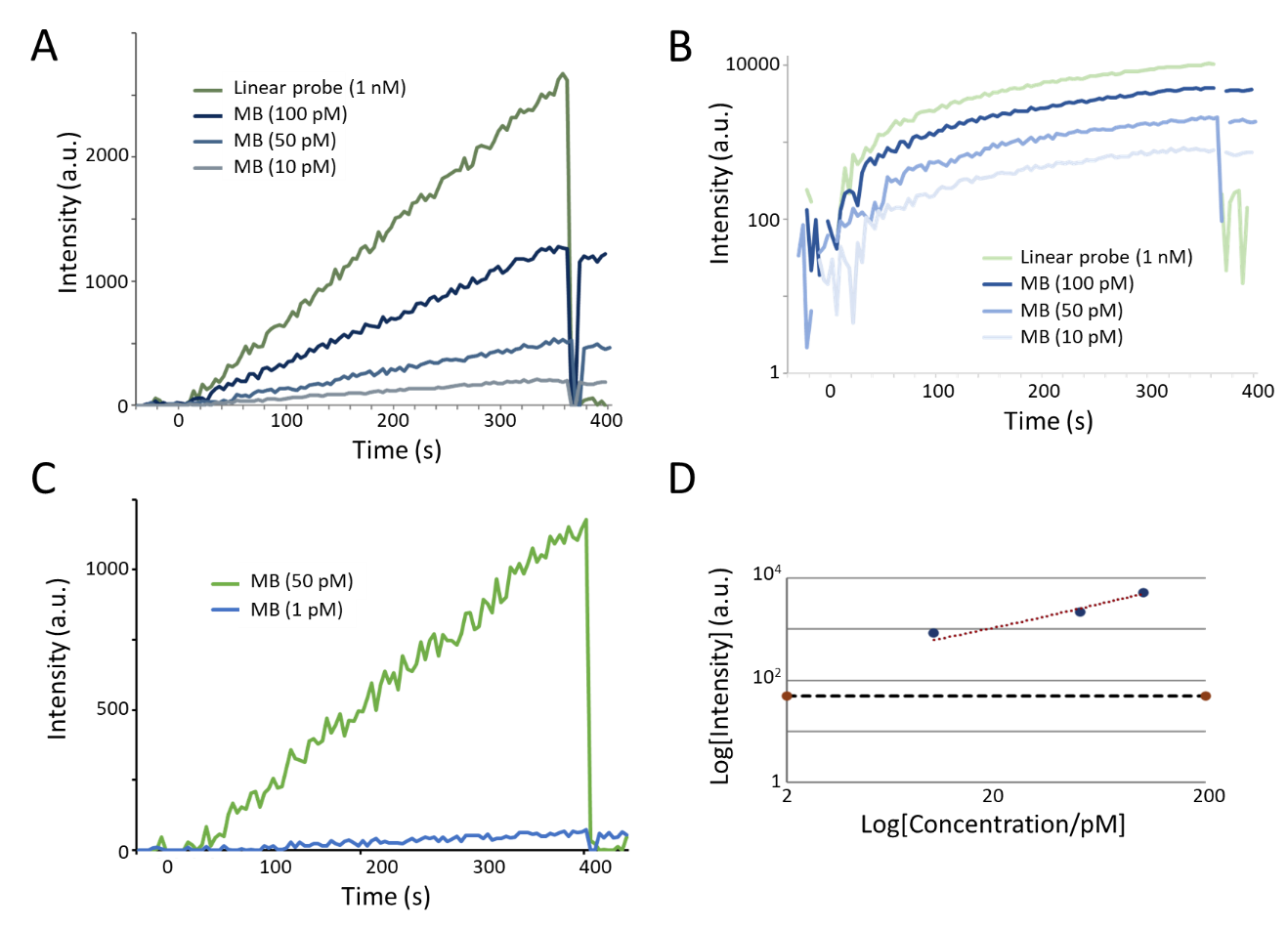


**Supplementary Figure S1: (A)** Maximum fluorescence intensity in the concentration module over time for various target concentrations, using either a linear probe or MB, as indicated in the legend. **(B)** Same data as in (A), with the y-axis plotted on a logarithmic scale. The rate of intensity increase is independent of probe concentration and primarily governed by the electrohydrodynamic behavior of the concentration module. **(C)** Fluorescence signal comparison at target concentrations of 1 pM and 50 pM. **(D)** Maximum fluorescence signal recorded at 300 s plotted against target concentration. The dashed line indicates the baseline signal in the presence of PVP solution.


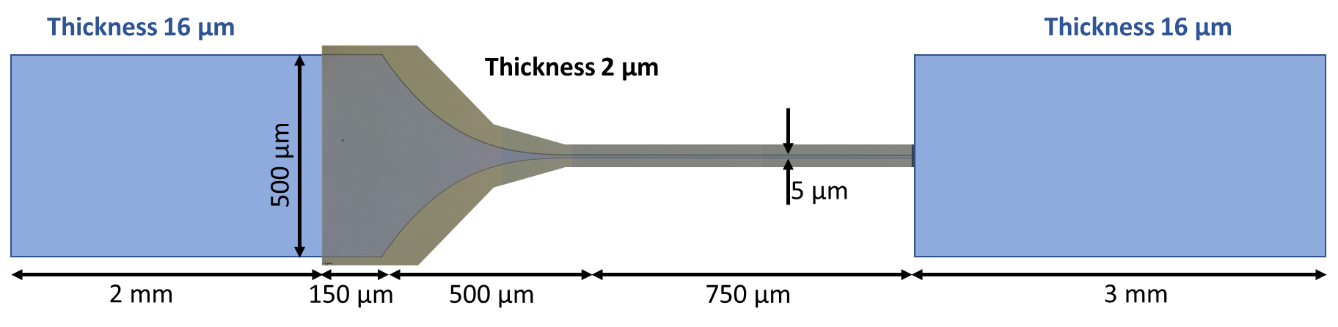


**Supplementary Figure S2:** The sketch presents the chip geometry. The funnel corresponds to the central section of 500 µm of the chip. The thick rectangular channels of 10 µm enable us to convey fluids to the constriction without excessive increase of the hydraulic resistance.


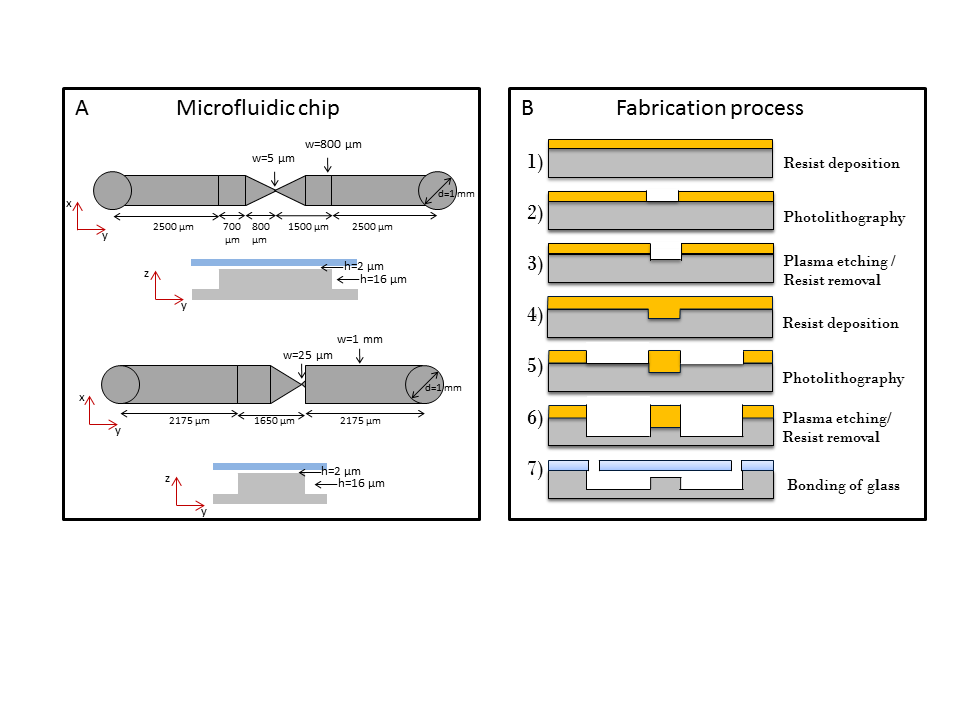


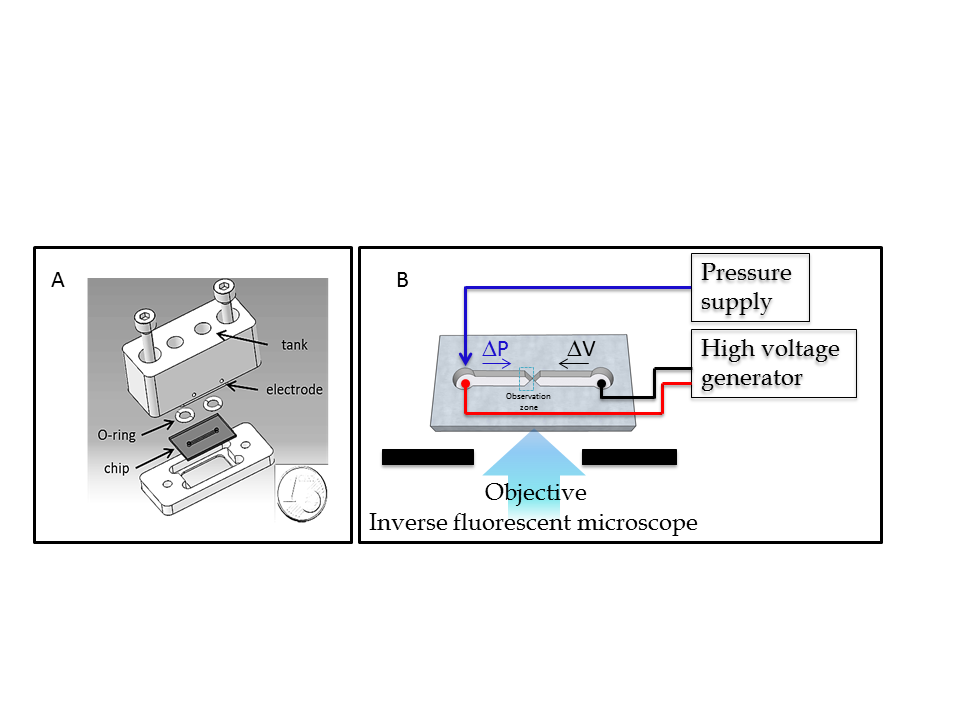


**D**

**C**

***Supplementary Figure S3: Microfluidic chip design, fabrication, and operation.* (*A)*** *Illustration of two microchannel designs represented in xy and zy planes. The glass slide is shown in blue in the transverse view, and silicon in gray.* ***(B)*** *Overview of the fabrication process. The positive resist (ECI 1.1 µm thickness) was spin coated on a silicon wafer (step 1). After photolithography and development of resist (step 2), the constriction pattern was dry-etched over 2 µm (step 3). Later, all steps were repeated (steps 4,5,6 in the illustration) to create straight channels which were dry-etched over 16 µm. The final step was to thermally grow a 300 nm layer of silicon oxide, and seal the water to a glass wafer by anodic bonding.* ***(C)*** *Schematic illustration of chip holder. The chip is placed on an aluminum support. The upper part of the holder contains inlet and outlet reservoirs (named tanks in image) interfaced with O-rings. This part is punched with two holes to insert platinum wires, which serve as electrodes.* ***(D*** *Operation of the chip on fluorescence microscope. The pressure is generated by coupling a pressure supply (Fluigent MFCS 7 bars, controlled with the manufacturer's software) directly into the support using pneumatic connectors (Legris, ref: 3171 04 20). The voltage is applied by a DC high voltage generator directly connected to the electrodes.*
